# Live-cell imaging of a plant virus replicase during infection using a genetically encoded, antibody-based probe

**DOI:** 10.1093/plphys/kiaf240

**Published:** 2025-06-06

**Authors:** Chiyusa Ishihara, Nobumitsu Sasaki, Yasuhiko Matsushita, Tomohiro Tsunoda, Tsutomu Arie, Richard S Nelson, Ken Komatsu

**Affiliations:** Graduate School of Agriculture, Tokyo University of Agriculture and Technology (TUAT), Fuchu, Tokyo 183-8509, Japan; Graduate School of Agriculture, Tokyo University of Agriculture and Technology (TUAT), Fuchu, Tokyo 183-8509, Japan; Institute of Global Innovation Research (GIR), Tokyo University of Agriculture and Technology (TUAT), Fuchu, Tokyo 183-8509, Japan; Graduate School of Agriculture, Tokyo University of Agriculture and Technology (TUAT), Fuchu, Tokyo 183-8509, Japan; Graduate School of Agriculture, Tokyo University of Agriculture and Technology (TUAT), Fuchu, Tokyo 183-8509, Japan; Graduate School of Agriculture, Tokyo University of Agriculture and Technology (TUAT), Fuchu, Tokyo 183-8509, Japan; Institute of Global Innovation Research (GIR), Tokyo University of Agriculture and Technology (TUAT), Fuchu, Tokyo 183-8509, Japan; Department of Entomology and Plant Pathology, Oklahoma State University, Stillwater, OK 74078, USA; Graduate School of Agriculture, Tokyo University of Agriculture and Technology (TUAT), Fuchu, Tokyo 183-8509, Japan; Institute of Global Innovation Research (GIR), Tokyo University of Agriculture and Technology (TUAT), Fuchu, Tokyo 183-8509, Japan

## Abstract

Replication of plant positive-strand RNA viruses occurs in association with intracellular membranes. To date, no versatile technology has been developed to directly label and visualize an active replicase in live plant cells because, in general, replicase function is not retained when it is fused to a protein for fluorescence imaging. We developed a technique to label and image a plant virus replicase during infection using the transiently expressed human influenza hemagglutinin (HA) frankenbody (FB), an antibody fragment that binds the HA epitope. The function of FB was demonstrated by visualizing the targeting of mCherry-fused FB (FB-mCherry) to an HA-tagged and GFP-fused endoplasmic reticulum (ER) marker protein in its native location. The combination of a two-component inducible system with the FB probe enabled FB-mCherry to label an HA epitope-tagged, functional replicase of Plantago asiatica mosaic virus (PlAMV) during its infection in *Nicotiana benthamiana* cells without affecting virus replication efficiency. The HA-tagged PlAMV replicase forms punctate structures associated with the ER and plasmodesmata and is localized in the vicinity of dsRNA, a hallmark of a viral replication complex. This application of epitope tag-binding intracellular probes for live subcellular imaging in plant cells offers insights into the localization dynamics of an active plant virus replicase during infection and the potential to co-localize host factors with the active replicase in situ.

## Teaser

A genetically encoded fluorescent probe based on an antibody fragment that binds to the hemagglutinin (HA) epitope enables live subcellular imaging of an HA-tagged functional plant virus replicase.

## Introduction

Positive-strand RNA ((+)RNA) viruses infect a wide range of hosts, including humans and plants, causing various diseases. Replication of these viruses begins with translation of virus replicase and auxiliary proteins from the genomic RNA. These proteins then localize to cellular membranes, induce their rearrangements, and recruit viral RNA and host factors to form the viral replication complex (VRC) or the viral replication organelle (VRO) (Ahlquist et al. 2003; Heinlein 2015; Ishibashi and Ishikawa 2016; Nagy and Feng 2021; Neufeldt and Cortese 2022; He et al. 2023; Jovanović et al. 2023). VRCs originate from different organelles including endoplasmic reticulum (ER), Golgi, mitochondria, chloroplasts, and peroxisomes, depending on virus species (Laliberté and Zheng 2014; Shulla and Randall 2016; He et al. 2023). Within VRCs, negative-strand RNA is synthesized using the viral genomic RNA as a template, resulting in double-stranded RNA (dsRNA) intermediates, followed by the synthesis of new positive strands. VRCs protect the viral genomic RNA from host antiviral defenses, enabling efficient replication (Ahlquist et al. 2003; Den Boon et al. 2010; Ishibashi and Ishikawa 2016; Nagy and Feng 2021). Understanding the processes of VRC formation by the viral replicase and its localization within the cell could lead to the development of novel technologies for plant virus control.

VRC localization in virus-infected plant cells has been studied using immunostaining with antibodies against replicase and dsRNA (Más and Beachy 1999; Bamunusinghe et al. 2009; Cotton et al. 2009; Kaido et al. 2014), as well as expressing whole or complementing dsRNA-binding proteins fused with a fluorescent reporter in transient expression or stable transformation (Cheng et al. 2015; Monsion et al. 2018; Wu and Nagy 2019; Wu et al. 2019; Takata et al. 2022). Live visualization of the dynamics of the replicase itself during VRC formation has been attempted but not achieved for many plant (+)RNA viruses (e.g. Wu et al. 2019). Previous studies visualized the replicase or auxiliary replication proteins by their fusion with a fluorescent protein. However, with the exception of a few successful cases, including a labeled NIb protein of turnip mosaic virus (TuMV) (Cheng et al. 2015), the majority of these studies have failed to demonstrate replication competence (Chen and Ahlquist 2000; Liu et al. 2005; Grangeon et al. 2013). The difficulty in direct labeling a replicase for visual detection during replication may be due to steric hindrances imposed by the fused fluorescent protein (van der Schaar et al. 2016). To date, there has been no general and versatile strategy for labeling the replicase of (+)RNA viruses.

As an alternative, an intracellular, single-chain variable fragment (scFv) antibody-based probe can be used, where antibody fragments are fused to fluorescent proteins to label target proteins (Shibuta et al. 2021). However, this method requires cloning of antibody genes for each target protein and demonstrating their functionality in living cells, making it challenging to apply to new targets. Therefore, other techniques have been developed to monitor the subcellular localization of epitope-tagged proteins using nanobodies or scFv antibodies that specifically bind to the epitope tag. These include NbALFA, a nanobody with high affinity for the 15-amino acid ALFA tag (Götzke et al. 2019), and SunTag, a multi-epitope scaffold that recruits multiple antibody fusions for amplified signals (Tanenbaum et al. 2014 ). Recently, a hybrid scFv called human influenza hemagglutinin (HA) frankenbody (referred to as FB in this article) has been developed (Zhao et al. 2019). HA FB binds the 9-amino acid HA epitope and can serve as a compact intracellular probe when fused with a fluorescent protein (Zhao et al. 2019). However, such epitope tag-binding intracellular probes have not been applied in plant cells.

Plantago asiatica mosaic virus (PlAMV), a (+)RNA virus of the genus *Potexvirus* in the family *Alphaflexiviridae*, has a genome that contains 5 open reading frames (ORFs), with ORF1 encoding the replicase (RNA-dependent RNA polymerase), ORF2, 3, and 4 encoding 3 movement proteins called triple gene block proteins 1, 2, and 3 (TGBp1, TGBp2, and TGBp3), and ORF5 encoding the coat protein (CP) (Komatsu and Hammond 2022). PlAMV is one of the most problematic pathogens infecting ornamental lilies, which frequently causes severe necrotic symptoms. Meanwhile, it has been used as a model plant RNA virus due to its efficient infection in the model plants *Arabidopsis thaliana* and *Nicotiana benthamiana* (Komatsu and Hammond 2022). The PlAMV replicase is a large protein of over 150 kDa containing methyltransferase (MET), helicase (HEL), and RNA polymerase (POL) domains. We recently found that the transient expression of the MET domain fused to a fluorescent protein induced small punctate structures and a large perinuclear complex, both of which are associated with ER (Komatsu et al. 2021). Although these structures were similar to those detected by immunostaining with dsRNA antibodies, which are thought to represent the VRC of PlAMV, it remains unclear whether they reflect the actual replicase localization during virus replication. Moreover, a replicase or its partial protein of other viruses of the same genus show different localization patterns depending on their expression context. In a study of potato virus X (PVX), a virus of the same genus as PlAMV, transient expression of a portion of the replicase fused to a fluorescent protein in the context of a virus infection resulted in the formation of granules at the entrance of plasmodesmata (PD) (Tilsner et al. 2013). Furthermore, a recent study showed that the full-length PVX replicase, fused to a fluorescent protein and not likely to be functional, localized to the nucleus when expressed alone, but formed irregular aggregates co-localized with dsRNA in the cytoplasm, including the perinuclear region, during virus infection (Wu et al. 2019). How these localization patterns relate to the position of a functional replicase during infection is not known.

Here we report an imaging approach using mCherry-fused FB, combined with an improved transactivation-based two-component expression strategy (Brand et al. 2006), to observe the subcellular location of an epitope-tagged, functional PlAMV replicase in live plant cells during viral infection. We visualized the HA-tagged PlAMV replicase forming punctate structures associated with the ER and PD. We also found that these structures were near dsRNA foci. This intracellular antibody-based imaging system provides insights into the localization dynamics of an actively replicating plant virus replicase during infection.

## Results

### FB-mCherry targets HA-tagged proteins in plant cells

**Figure 1.**
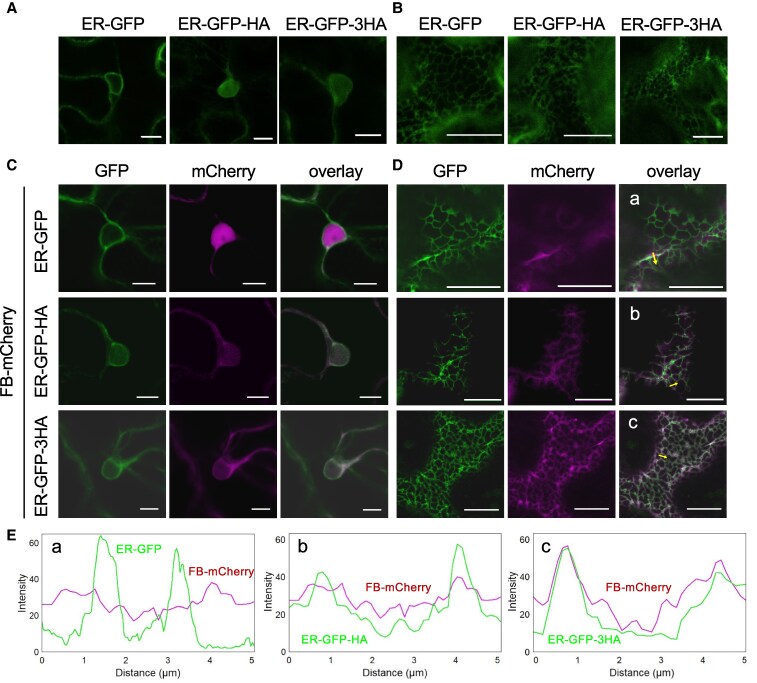
HA frankenbody (FB) is targeted to HA-tagged endoplasmic reticulum (ER)-GFP *in planta*. **A-B)** Confocal images of the subcellular localization of ER-GFP, ER-GFP-HA, or ER-GFP-3HA, which have 0, 1, or 3 HA tags fused with ER-GFP, respectively, transiently expressed in *Nicotiana benthamiana* leaves. Images were taken at 48 h post-infiltration (hpi). Images were taken at a perinuclear A) and a peripheral B) plane. Scale bars, 10 *µ*m (A) and 20 *µ*m (B). **C-D)** Confocal images of the subcellular localization of FB fused with mCherry (FB-mCherry) co-expressed with ER-GFP, ER-GFP-HA, or ER-GFP-3HA, taken at a perinuclear (C) and a peripheral (D) plane at 48 hpi. Scale bars, 10 *µ*m (C) and 20 *µ*m (D). **E)** Fluorescence intensities of GFP and mCherry along the arrow in the overlay images in (D, a-c).

In this study, we used FB-mCherry, in which mCherry was fused to the C-terminus of FB, to investigate whether it could label HA-tagged proteins in plant cells (Zhao et al. 2019). As a proof of concept, we first tested whether FB-mCherry targeted an HA-tagged ER-localized GFP fusion protein (ER-GFP) (Nelson et al. 2007). We generated ER-GFP-HA and ER-GFP-3HA, in which 1 and 3 HA tags, respectively, were added to ER-GFP. All constructs used in this assay were expressed under the control of the constitutive cauliflower mosaic virus 35S promoter. Transient expression of ER-GFP-HA and ER-GFP-3HA in *Nicotiana benthamiana* leaves and confocal laser scanning microscopy (CLSM) showed perinuclear and reticulate network signals characteristic of ER localization, indicating that the addition of the HA tag does not alter the localization pattern of ER-GFP (Figs. 1, A and B). When we co-expressed FB-mCherry with ER-GFP-HA or ER-GFP-3HA, the localization patterns of GFP did not considerably differ from those of cells expressing the ER-GFP constructs alone (Figs. 1, C and D). In contrast, the subcellular localization pattern of FB-mCherry changed depending on the co-expressed construct. FB-mCherry fluorescence was observed in the nucleus and cytoplasm when co-expressed with ER-GFP lacking the HA tag (Fig. 1C). On the other hand, when co-expressed with ER-GFP-HA or ER-GFP-3HA, FB-mCherry fluorescence was mainly observed in the perinuclear ER, although some fluorescence was also observed in the nucleus (Fig. 1C). On the peripheral cell surface, when co-expressed with the ER-GFP without the HA tag, the fluorescence of FB-mCherry was localized in the cytoplasm and did not coincide with the GFP fluorescence representing the ER network (Fig. 1D). However, when co-expressed with ER-GFP-HA or ER-GFP-3HA, the fluorescence of FB-mCherry merged with the reticulate network pattern of GFP fluorescence (Fig. 1D). The co-localization of FB-mCherry fluorescence with ER-GFP-3HA tends to be more prominent than that with ER-GFP-HA, as shown by signal intensity measurement (Fig. 1E). These results indicate that FB-mCherry targeted to HA-tagged proteins and changed its subcellular location according to that of the targeted proteins. In conclusion, this experiment confirmed the potential of FB-mCherry to study the intracellular localization of HA-tagged proteins in living plant cells.

### Subcellular localization of FB-mCherry expressed under the control of the 35S promoter does not change upon co-expression with an HA-tagged PlAMV replicase

**Figure 2.**
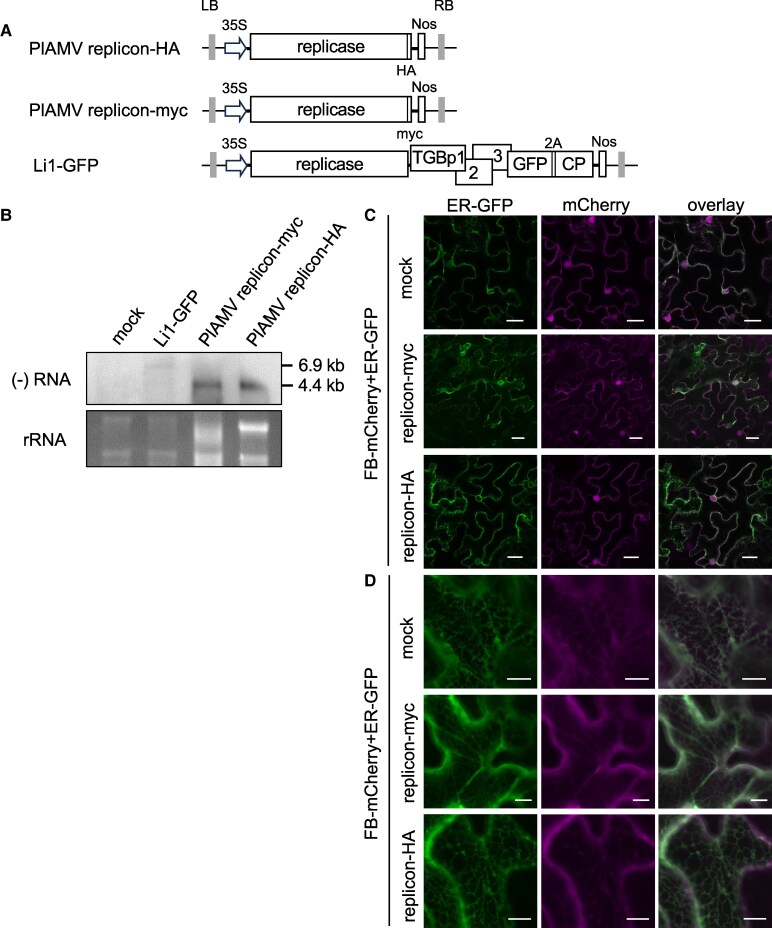
FB-mCherry expressed under the control of the 35S promoter does not change its subcellular localization upon co-expression with an HA-tagged PlAMV replicase. **A)** Schematic representations of the construct that launches Li1-GFP or PlAMV replicon infection by agroinfiltration. Li1-GFP is GFP-encoding PlAMV (Minato et al. 2014). PlAMV replicon-HA and PlAMV replicon-myc (Komatsu et al. 2011) have the replicase gene only, flanked by untranslated regions required for virus replication. In Li1-GFP, TGBp1, 2, and 3 represent triple gene block proteins that are involved in viral cell-to-cell movement, and CP represents coat protein. GFP was fused to the N-terminus of the CP via the foot-and-mouth disease virus 2A peptide. 35S represents the cauliflower mosaic virus 35S promoter, and Nos represents the nopaline synthase terminator. LB and RB indicate the left and right borders of the T-DNA sequence, respectively. **B)** Northern blotting analysis of the negative-strand RNA of PlAMV or its replicons in *Nicotiana benthamiana* leaves at 72 h post-infiltration (hpi) of the virus construct shown in (A). Approximately 1.5 *µ*g of total RNA was loaded and the digoxigenin (DIG)-labelled RNA probe targeting the negative-strand of the replicase-coding region was used. Total RNA extracted from uninfiltrated leaves (mock) served as a negative control, while Li1-GFP and PlAMV replicon-myc served as positive controls. Ethidium bromide-stained ribosomal RNA (rRNA) is shown as a loading control. **C-D)** Confocal images of the subcellular localization of endoplasmic reticulum (ER)-GFP and FB-mCherry without virus construct (mock), or with PlAMV replicon-myc or PlAMV replicon-HA in agroinfiltrated *N. benthamiana* leaves at 48 hpi. C) shows transverse sections of whole epidermal cells, and D) shows the ER network on the cell peripheral surface. Scale bars, 25 *µ*m (C) and 10 *µ*m (D).

We have previously reported that a PlAMV replicon, which has only the replicase-encoding ORF with the flanking untranslated regions, could replicate efficiently even when its replicase was fused with a c-Myc tag at the C-terminus (PlAMV replicon-myc) (Komatsu et al. 2011; Fig. 2A). Based on this previous finding, we added an HA tag instead of the c-Myc tag to the C-terminus of the replicase in the PlAMV replicon (PlAMV replicon-HA, Fig. 2A) to visualize the subcellular location of the potentially functional HA-tagged replicase during replication using FB-mCherry. After agroinfiltration of PlAMV replicon-HA, we performed northern blotting using an RNA probe targeting viral negative-strand RNA, a marker of viral replication. At 3 days post-infiltration (dpi), full-length negative-strand genomic RNA was detected from PlAMV replicon-HA infiltrated leaves, similar to PlAMV replicon-myc and GFP-expressing PlAMV as controls (Li1-GFP) (Fig. 2B), indicating that the addition of an HA tag still allows replication of PlAMV. Furthermore, similar to our previous report for the PlAMV replicon-myc (Komatsu et al. 2011), we observed necrosis in the almost entire part of the leaf infiltrated with PlAMV replicon-HA at 3 dpi, suggesting that the HA-tagged replicase was expressed in a substantial number of cells within the infiltrated area.

To investigate the subcellular location of the replicase in the context of virus replication, PlAMV replicon-HA was co-expressed with FB-mCherry and the ER marker ER-GFP in *N. benthamiana*, followed by CLSM at 48 h post-infiltration (hpi) when necrosis was not yet observed. However, even when co-expressed with PlAMV replicon-HA, FB-mCherry was localized throughout the cytoplasm and nucleus, and this localization pattern was similar to that when FB-mCherry was expressed alone or co-expressed with PlAMV replicon-myc (Figs. 2, C and D). A similar localization of FB-mCherry in the cytoplasm and nucleus was observed in GFP-expressing cells infected with Li1-replicase-HA-GFP, which encodes an HA-tagged replicase and expresses GFP as a fusion to the N-terminus of the CP via the self-cleaving 2A peptide of foot-and-mouth disease virus (Supplementary Fig. S1). This result suggests that the nucleocytoplasmic localization pattern of FB-mCherry remains unchanged, regardless of the presence or absence of virus-encoded proteins other than the replicase (i.e. TGBp1-3 and CP). Previous studies reported that, when transiently expressed as a fusion with a fluorescent protein, the subcellular localization of replicases of potexviruses, including PlAMV, is closely associated with the ER (Bamunusinghe et al. 2009; Komatsu et al. 2021). Taken together, these results suggest that the localization of the HA-tagged replicase during viral replication cannot be observed by the FB probe in this experimental condition.

### Development of a two-component system that enables replication-dependent expression of FB-mCherry

**Figure 3.**
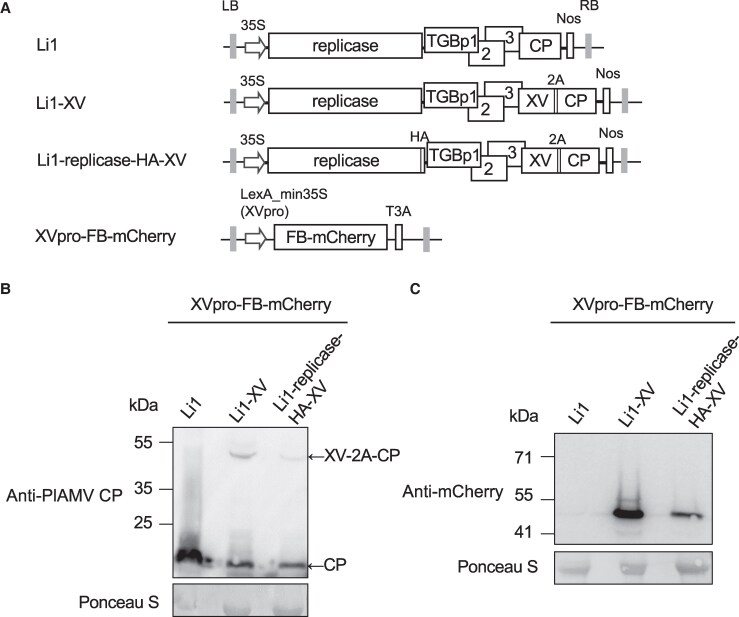
A two-component inducible system that enables the PlAMV replication-dependent expression of FB-mCherry. **A)** Schematic representations of infectious clones of wild-type PlAMV (Li1) and its variants expressing XV transcription factor, and XVpro-FB-mCherry construct. In Li1-XV and Li1-replicase-HA-XV, an artificial chimeric transcriptional activation factor XV was fused to the N-terminus of the coat protein (CP) via the foot-and-mouth disease virus 2A peptide. Triple gene block proteins 1-3 (TGBp1-3) represent movement proteins. LexA_min35S represents the XV-dependent promoter (XVpro), and T3A denotes the T3A terminator. See the legend of Fig. 2A for other abbreviations. **B-C)** Immunoblotting analyses of PlAMV CP (and XV-2A-CP) and FB-mCherry in *Nicotiana benthamiana* leaves co-expressing XVpro-FB-mCherry and either Li1, Li1-XV, or Li1-replicase-HA-XV at 48 h post-infiltration (hpi). Ponceau S-stained Rubisco large subunit serves as a loading control.

One of the most plausible explanations for the nuclear and cytoplasmic localization of FB-mCherry upon co-expression with the PlAMV replicon-HA in the above experiment could be attributed to the higher accumulation of FB-mCherry, which is produced by the 35S promoter, compared to the limited accumulation of HA-tagged replicase, which is strongly dependent on viral replication (Komatsu et al. 2011). We hypothesized that a significant portion of the observed fluorescence in the nucleus and cytoplasm was due to the high abundance of free FB-mCherry protein, which exceeded the level needed to bind to the available HA-tagged replicase. To reduce the background fluorescence of the free FB-mCherry, we attempted to express FB-mCherry in a virus replication-dependent, inducible manner by taking advantage of the chimeric transcription factor XVE, which is used in the two-component, estradiol-dependent inducible system (Zuo et al. 2000; Brand et al. 2006). In our inducible system, an XV domain lacking the estrogen receptor domain (E) of XVE was used and its coding sequence was inserted into the PlAMV vector to be produced from a subgenomic RNA (Minato et al. 2014) (Fig. 3A). For XV-dependent expression of FB-mCherry, it was cloned downstream of the XV binding promoter (LexA_min35S; hereafter called XVpro) of the vector pMDC221 (Brand et al. 2006). In this system, the subgenomic RNA is transcribed concomitantly with viral replication, leading to XV synthesis only in cells in which PlAMV replicates. We expected that this tightly controlled expression of FB-mCherry coupled with virus replication could suppress excessive background fluorescence of free FB-mCherry.

We generated 2 viral constructs: Li1-XV encoding XV fused N-terminally to the PlAMV CP via the 2A peptide of foot-and-mouth disease virus, and Li1-replicase-HA-XV, a Li1-XV derivative encoding the HA-tagged replicase (Fig. 3A). Each virus construct was co-expressed with XVpro-FB-mCherry in *N. benthamiana*, and total protein was extracted at 2 dpi. Immunoblotting analysis using PlAMV CP antibody confirmed the accumulation of CP from both Li1-XV and Li1-replicase-HA-XV, indicating that these viruses are capable of replication. In addition, a slowly migrating band corresponding to the intact XV-2A-CP fusion, not cleaved by the 2A peptide, was also detected from both viruses, indirectly indicating that XV is expressed by the infection of these viruses (Fig. 3B). To confirm that FB-mCherry was induced in an XV-dependent manner, we performed immunoblotting analysis using mCherry antibody. While FB-mCherry was not detected in leaves infected by Li1, it was detected in leaves infected by Li1-XV or Li1-replicase-HA-XV (Fig. 3C). Taken together, the two-component system using XV-encoding PlAMV constructs and XVpro-FB-mCherry allowed the replication-dependent expression of FB-mCherry and thus was used for subsequent analyses.

### Replication-dependent expression of FB-mCherry results in fluorescent granule formation in cells infected by PlAMV encoding an HA-tagged replicase

**Figure 4.**
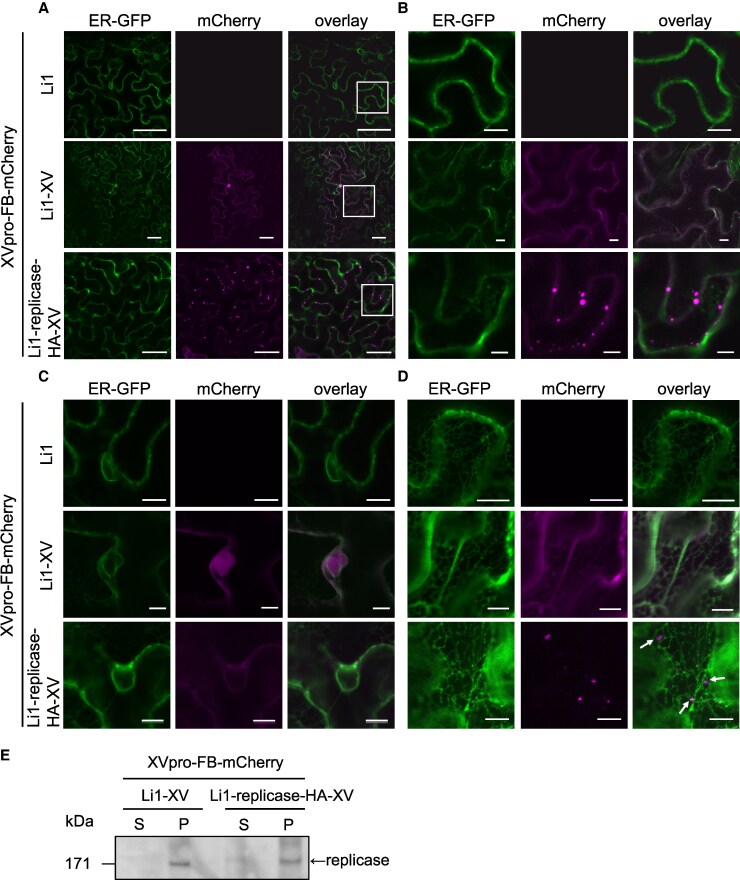
XV-dependent expression of FB-mCherry enables labeling of an HA-tagged replicase, which forms granules along the cell membrane and on the peripheral cell surface. **A-D)** Confocal images of the subcellular localization of FB-mCherry in leaves of *Nicotiana benthamiana* line 16c that expresses endoplasmic reticulum (ER)-GFP, co-expressing XVpro-FB-mCherry and Li1, Li1-XV, or Li1-replicase-HA-XV at 48 h post-infiltration (hpi). **A)** shows a transverse section of the whole epidermal cells and **B)** shows magnified images of the area in the white squares in A). **C)** shows the perinuclear region and **D)** shows the ER network on the cell peripheral surface. The arrows in the overlay picture of **D)** indicate FB-mCherry fluorescence adjacent to the ER network. Scale bars, 50 *µ*m in (A), and 10 *µ*m in (B-D). **E)** Immunoblotting analysis of PlAMV replicase using PlAMV replicase antibody in *N. benthamiana* leaves co-expressing XVpro-FB-mCherry and Li1-XV or Li1-replicase-HA-XV at 48 hpi. Total protein was separated into soluble (S) and membrane-enriched pellet (P) fractions.

Using the two-component XV inducible system, we attempted to observe the subcellular location of the HA-tagged replicase of PlAMV. Because a transiently expressed replicase of potexviruses has been reported to be closely associated with the ER, we co-expressed Li1, Li1-XV, or Li1-replicase-HA-XV with XVpro-FB-mCherry in *N. benthamiana* line 16c, which expresses ER-localized GFP (Ruiz et al. 1998). CLSM observation was performed at 48 hpi. No FB-mCherry fluorescence was observed in cells infected by Li1 (Figs. 4A to 4D). On the other hand, FB-mCherry fluorescence was evenly distributed within the nucleus and throughout the cytoplasm of cells infected by Li1-XV (Figs. 4A to 4D). This localization pattern was similar to that of FB-mCherry expressed from the 35S promoter (Fig. 2C). In contrast, in leaves infected by Li1-replicase-HA-XV, FB-mCherry granules with diameters of approximately 1.0 to 5.0 *μ*m were observed along the cell periphery, while FB-mCherry fluorescence was also observed occasionally in the nucleus and cytoplasm (Figs. 4A to 4C). In addition, relatively small granules of FB-mCherry, approximately 0.5 to 2.5 *μ*m in diameter, were observed at the peripheral cell surface, mostly adjacent to the ER network (Fig. 4D). Thus, significantly different patterns of FB-mCherry localization were observed between Li1-XV- and Li1-replicase-HA-XV-infected leaves. Furthermore, we confirmed by immunoblotting analysis that the accumulation levels of the replicase of Li1-XV and Li1-replicase-HA-XV in the membrane-enriched pellet fraction (P) were comparable (Fig. 4E). These collective observations suggested that the HA-tagged replicase of PlAMV, visualized by FB-mCherry, forms granules along the cell membrane and on the peripheral cell surface, which are closely associated with the ER.

### FB does not affect accumulation of the virus encoding an HA-tagged replicase

**Figure 5.**
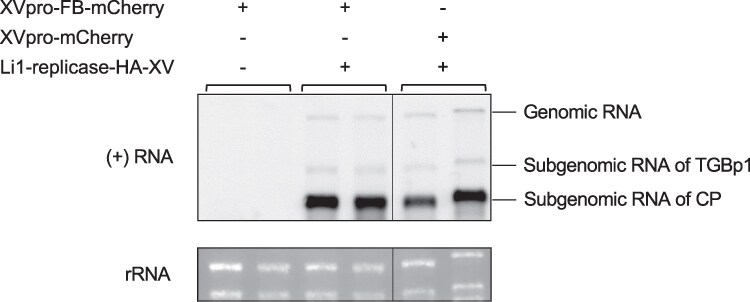
XV-dependent expression of FB-mCherry does not affect accumulation of PlAMV encoding an HA-tagged replicase. Northern blotting of PlAMV genomic and subgenomic RNAs in *Nicotiana benthamiana* leaves co-expressing Li1-replicase-HA-XV with XVpro-FB-mCherry or XVpro-mCherry at 48 h post-infiltration (hpi). Approximately 1 *µ*g of total RNA, each extracted from 2 independent leaves, was loaded and digoxigenin-labeled RNA probe targeting the coat protein (CP)-encoding region of the positive-strand of PlAMV was used. Total RNA extracted from leaves expressing only XVpro-FB-mCherry served as a negative control. Ethidium bromide-stained ribosomal RNA (rRNA) was served as a loading control.

Our CLSM observations revealed significant differences in the subcellular location of FB-mCherry in leaves infected with Li1-replicase-HA-XV and Li1-XV, suggesting that FB-mCherry binds to the HA-tagged replicase. We next investigated whether FB affected accumulation of the virus with an HA-tagged replicase. Total RNA was extracted at 48 hpi from *N. benthamiana* leaves co-expressing Li1-replicase-HA-XV with XVpro-FB-mCherry or XVpro-mCherry and analyzed by northern blotting with a probe targeting the *CP* region of the PlAMV genome. The accumulation of genomic RNA and 2 subgenomic RNAs of Li1-replicase-HA-XV in leaves expressing FB-mCherry was comparable to that in leaves expressing free mCherry, which does not target the HA tag (Fig. 5). This result indicated that targeting of FB to the HA tag fused to the PlAMV replicase does not affect virus replication.

### FB-mCherry granules formed during Li1-replicase-HA-XV infection are located in close proximity to PD and dsRNA granules

**Figure 6.**
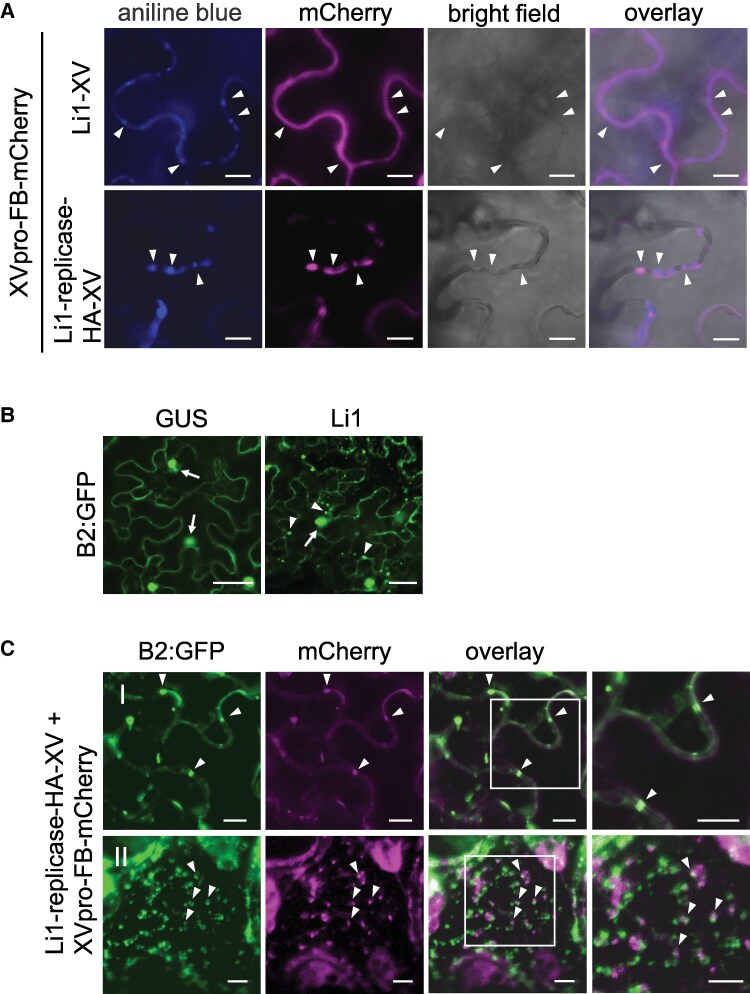
Granules of HA-tagged replicase labeled with FB-mCherry are localized in close proximity to plasmodesmata and dsRNA. **A)** Confocal images of leaves of *Nicotiana benthamiana* co-expressing XVpro-FB-mCherry and Li1-XV or Li1-replicase-HA-XV at 48 h post-infiltration (hpi), stained with aniline blue for 30 min before observation. Arrowheads represent location of plasmodesmata, which is shown by fluorescence of aniline blue staining and FB-mCherry. Scale bar, 25 *µ*m. **B)** Confocal images of B2:GFP *N. benthamiana* leaves expressing a control β-glucuronidase (GUS) (left) or Li1 (right) at 48 hpi. Arrows indicate the nucleus and arrowheads indicate granules of B2:GFP at the cell periphery. Scale bar, 25 *µ*m. **C)** Confocal images of the subcellular localization of FB-mCherry in B2:GFP *N. benthamiana* leaves co-expressing Li1-replicase-HA-XV and XVpro-FB-mCherry at 48 hpi. (I) represents a transverse section of the cell, while (II) represents the cell peripheral surface region. The 2 images on the far right show magnified images of the area in the white squares in the overlay images. Arrowheads represent FB-mCherry fluorescence proximal to or co-localized with B2-GFP fluorescence that indicates double-stranded RNA. Scale bar, 10 *µ*m.

Since small granular structures of FB-mCherry were observed at the peripheral cell surface during replication of Li1-replicase-HA-XV (Fig. 4D), we hypothesized that the FB-mCherry granules might be closely associated with PD. To investigate the detailed subcellular localization of FB-mCherry, we performed aniline blue staining, which labels the callose accumulation at PD. FB-mCherry granules in the cells infected by Li1-replicase-HA-XV were found to be in close proximity or colocalized with aniline blue fluorescence at 48 hpi (Fig. 6A). In contrast, FB-mCherry fluorescence did not co-localize with aniline blue fluorescence in the cells infected by Li1-XV (Fig. 6A). These results indicated that PlAMV replicase could localize near the PD during virus replication.

To elucidate whether the FB-mCherry granules observed during the replication of PlAMV encoding an HA-tagged replicase represent VRCs, we next analyzed the co-localization of FB-mCherry-labeled replicase with dsRNA, an intermediate of virus replication. We used *N. benthamiana* plants expressing B2:GFP, which can label dsRNA with GFP fluorescence (Monsion et al. 2018). We firstly tried to confirm whether PlAMV infection induces dsRNA granules in the B2:GFP plant, as shown previously for PVX (Monsion et al. 2018). At 48 hpi of Li1 to B2:GFP plants, GFP fluorescence was observed in the nucleus, with numerous granules also observed at the cell periphery and in the cytoplasm, which were rarely observed in leaves expressing β-glucuronidase (GUS, negative control) (Fig. 6B). When Li1-replicase-HA-XV and XVpro-FB-mCherry were co-expressed in B2:GFP plants, FB-mCherry granules were found proximal to B2:GFP fluorescence along the cell periphery in transverse sections at 48 hpi (Fig. 6C, panel I). In addition, FB-mCherry fluorescence was associated with B2:GFP fluorescence at the peripheral cell surface layer (Fig. 6C, panel II). In conclusion, the FB-mCherry granules observed at the cell periphery upon expression of an HA-tagged replicase were associated with or proximal to dsRNA, suggesting that FB-mCherry can label the HA-tagged replicase associated with VRC.

## Discussion

In this study, we demonstrate that an antibody-based probe combined with our inducible XV system enables the observation of the low-abundance replicase of plant viruses in the context of viral infection. This study represents the application of FB for live-cell imaging *in planta*. In a previous study, attempts have been made to use a GFP-binding protein (GBP) derived from a single-chain antibody from *Camelidae* sp. (chromobody) for live-cell imaging *in planta*. However, the chromobody altered the localization and function of target GFP-fused proteins (Schornack et al. 2009). In contrast to the GFP-binding chromobody, we have successfully exploited the binding property of FB to the small HA epitope, which is unlikely to alter the localization or inhibit the function of its target HA-tagged proteins. Indeed, HA-tagged ER-GFP showed the same ER network pattern regardless of the labeling with FB-mCherry (Fig. 1). In addition, the accumulation level of the PlAMV variant encoding replicase-HA (Li1-replicase-HA-XV) in the leaves expressing FB-mCherry was comparable to that in the leaves expressing a control free mCherry (Fig. 5). These results clearly show that binding of FB to HA does not affect ER localization of ER-GFP-HA or the replicase activity of PlAMV replicase-HA. Therefore, FB-mCherry or any fluorescently labeled FB fusions can be a versatile tool for live-cell imaging of an HA-tagged protein *in planta*.

It should be noted, however, that successful application of the FB technology may require an appropriate balance between the amounts of FB and its target protein. Our initial attempt to express FB-mCherry under the control of the constitutive 35S promoter to visualize HA-tagged replicase during viral replication resulted in a nuclear and cytoplasmic localization pattern indistinguishable from that of FB-mCherry expressed alone (Fig. 2C). This pattern was not consistent with previous works reporting that the potexvirus replicase is associated with ER (Bamunusinghe et al. 2009; Komatsu et al. 2021). We interpreted this result as the excessive background fluorescence of untargeted or unbound FB-mCherry in the cytoplasm and nucleus, due to its high expression by the 35S promoter, making it difficult to detect the specific localization of replicase-HA labeled by FB-mCherry. This interpretation is supported by the finding that the HA-tagged replicase was observed near the ER networks when FB-mCherry was expressed by the inducible XV system using Li1-replicase-HA-XV in a virus replication-dependent manner (Fig. 4D). Therefore, the expression level of FB should be optimized to detect the specific localization of HA-tagged target proteins, and the two-component inducible XV system developed in this study is effective in achieving this goal.

Additionally, the specificity and intensity of labeling by FB-mCherry may be affected by the number of HA tags fused to the protein of interest, as suggested by our observation of ER-associated FB-mCherry fluorescence co-expressed with ER-GFP-3HA or ER-GFP-HA (Figs. 1C-1E). Indeed, in a study of animal cells, increasing the number of HA tags resulted in enhanced epitope-specific fluorescence labeled by FB (Zhao et al. 2019). In our study, however, only one HA tag was fused to PlAMV replicase to avoid the potential negative effect of multiple tags on replicase activity. In future experiments, the localization of replicase will be detected more easily and clearly by increasing the number of HA tags.

In this study, viral replication-dependent expression of FB-mCherry revealed the localization of replicase-HA as granules proximal to the ER network at the cell surface (Fig. 4D) and to the PD along the transverse cell periphery (Fig. 6A). This result indicates that PlAMV replicase forms granular structures associated with the ER and PD during replication. Our observation is consistent with the previous findings that full-length and partially truncated replicase of PVX, a member of the same genus *Potexvirus* as PlAMV, were observed as granules localized closely to the ER network and aligned with the PD, respectively, when transiently expressed alone (Bamunusinghe et al. 2009; Tilsner et al. 2013). Thus, PlAMV replicase-HA retains the common subcellular localization property of the potexvirus replicase. Additionally, our CLSM imaging of B2:GFP plants showed that the infection with a wild-type PlAMV (Li1) and its variants (Li1-replicase-HA-XV) caused the formation of B2:GFP granules (Fig. 6), indicating that dsRNA clusters were generated upon virus infection. Since specific granular localization of GFP fluorescence was minimally observed in GUS-expressing control leaves of B2:GFP plants (Fig. 6B), we have assumed that the dsRNA clusters, shown by B2:GFP granules in virus-infected leaves, represent genomic dsRNA intermediates produced by virus replication. Importantly, we also found that FB-mCherry granules formed by Li1-replicase-HA-XV were co-localized or adjacent to the B2:GFP granules (Fig. 6C). This close alignment of the granules of FB-mCherry and B2:GFP may indicate that each FB-mCherry granule represents a replicase-HA-containing VRC or replication-related site. It is also worth noting that not all B2:GFP granules appear to be proximal to or co-localized with FB-mCherry granules. This may be due to the lower labeling sensitivity of HA-tagged replicases by FB-mCherry compared to dsRNA by B2:GFP, which may be caused by the number and position of the epitope; only one HA epitope at the C-terminus of replicase can be bound by FB-mCherry, whereas the whole viral dsRNA molecule can be recognized by B2:GFP. Future studies are needed to improve the labeling sensitivity of the HA-tagged replicase, for example by increasing the number of HA epitope tags mentioned above or by using brighter fluorescent proteins than mCherry. Alternatively, the possibility cannot be excluded that B2:GFP granules not proximal to or co-localized with FB-mCherry granules are derived from siRNA bodies, which have been reported to be involved in antiviral RNA silencing (Tan et al. 2023).

As discussed above, our FB-mCherry-mediated observation of replicase-HA demonstrated that PlAMV replicase is associated not only with the ER but also with the PD during virus replication. The association of replicase with the ER network would be essential for virus replication, as ER membranes are thought to be a platform for the generation of VRCs of potexviruses, including PlAMV (Bamunusinghe et al. 2009; Komatsu et al. 2021). Meanwhile, the association of replicase with PD may be important for efficient intercellular transport of genomic RNAs immediately after virus replication. In support of this idea, localization of infection-specific bodies containing the tobacco mosaic virus replicase to the PD near the infection front has been reported (Szécsi et al. 1999). Studies on other viruses also provide evidence that replication and intracellular transport are spatially coupled at PD (Levy and Tilsner 2020). Additionally, a recent study of red clover necrotic mosaic virus (RCNMV) using B2:GFP plants showed that B2:GFP-labeled dsRNA clusters, which indirectly indicated the presence of the virus replicase, were relocated from the ER network to the vicinity of the PD over time after virus infection (Takata et al. 2022). Future spatiotemporal analysis using our live imaging method, in combination with the B2:GFP-mediated dsRNA observation, will reveal further details on the process of transition of replicase-containing granules from ER to PD during virus replication.

In our previous immunological analysis of a c-Myc-tagged replicase encoded by a PlAMV replicon, ER-associated granules of the replicase were detected around the nucleus in the infected *N. benthamiana* protoplasts (Komatsu et al. 2021). Similarly, we observed an ER-associated, large perinuclear complex of a GFP-fused MET domain of the PlAMV replicase, when expressed transiently in *N. benthamiana* leaves (Komatsu et al. 2021). In a recent study of bamboo mosaic virus, a member of the genus *Potexvirus*, its full-length replicase, fused with a fluorescent protein, was shown to form complexes at the periphery of the nucleus and overlap with dsRNA clusters visualized by B2:GFP (Lee et al. 2022). Therefore, it is possible that the potexvirus replicase coalesces at the perinuclear ER to form complexes in the virus infection process. However, in this study, the granules of FB-mCherry and B2:GFP were rarely observed around the nucleus in *N. benthamiana* infected with Li1-replicase-HA-XV and perinuclear labeling was minimal (Fig. 4C), suggesting that PlAMV replicase does not aggregate significantly in the perinuclear ER during replication. However, it is still possible that PlAMV replicase forms large perinuclear VRC during its natural infection context, which can be observed at different time points after the onset of virus infection. Since replicase-HA is functional in virus replication regardless of the presence of FB-mCherry (Fig. 5), the massive complexes of PlAMV replicase around the nucleus may not be needed for virus replication. Further analysis is required to elucidate the biological significance of the perinuclear complex of PlAMV replicase.

A limitation of this system is that we cannot analyze the very early stage of replication (before 24 hpi). Since the Agrobacterium-mediated gene delivery method is used for both virus infection and FB-mCherry expression, approximately half a day is inevitably required for the onset of virus replication to generate genomic and subgenomic RNA, the latter being the template for the synthesis of XV that activates the production of FB-mCherry. In addition, defense responses against Agrobacterium infection are shown to affect virus infection (Pruss et al. 2008). In fact, both FB-mCherry and B2:GFP granules were detected in a very limited number of cells at 24 hpi, when viral RNA was barely detectable by northern blotting (Supplementary Fig. S2). In contrast, microprojectile bombardment of a PVX infectious clone and mechanical inoculation of infectious RNA transcripts of RCNMV (Monsion et al. 2018; Takata et al. 2022) resulted in the early detection of virus dsRNA granules in B2:GFP-expressing *N. benthamiana* (6 hpi for PVX and 5 hpi for RCNMV). We are now generating transgenic *N. benthamiana* expressing FB-mCherry under the regulation of the XV promoter for the direct inoculation with an infectious clone Li1-replicase-HA-XV. A further issue to be addressed in future studies is that the antibody-based labeling method for detecting virus replicase in plant cells requires the expression of the XV transcription factor, which is dependent on virus replication. In the present study, XV is expressed from a subgenomic RNA, but how XV can be expressed depends on genome organization and gene expression strategy of each target virus and should be considered individually. To detect virus replicase at an early stage, it is necessary to express an appropriate amount of FB-mCherry before virus replication, which is a prerequisite for XV expression in this study. Alternative methods of expression, such as chemical- or light-inducible methods, which can induce an antibody-based probe are promising candidates to achieve this in an XV-independent manner (Omelina et al. 2022). With future improvements, we expect that the antibody-based probe system will facilitate more effective and accurate analysis of the localization and dynamics of plant and microbial proteins, thereby revealing the mechanisms underlying plant physiological processes, development, and defense responses against pathogens.

## Materials and methods

### Plant materials, growth conditions, and agroinfiltration

Wild-type and transgenic *Nicotiana benthamiana* plants were grown in Nippi Engei Baido no.1 soil (Nihon Hiryo, Tokyo, Japan) under long-day conditions (25 °C, 16-h light/8-h dark cycles) in a growth chamber. Transgenic *N. benthamiana* lines expressing an ER-localized GFP (line 16c) and a GFP-tagged dsRNA binding protein (B2:GFP) were provided by Dr. David Baulcombe (Ruiz et al. 1998) and Dr. Christophe Ritzenthaler (Monsion et al. 2018), respectively. Plants at 4 to 5 weeks after sowing were used for agroinfiltration, as described previously, with *Agrobacterium tumefaciens* strain EHA105 (Komatsu et al. 2021). OD_600_ values of each Agrobacterium suspension before mixing the same volume were 0.1 in Figs. 1, 2, C and D, 3, B and C, 4A to D, 5 and 6 and Supplementary Figs. S1 and S2, 0.5 in Fig. 2B, 0.3 in Fig. 4E, and 0.3 only for XVpro-FB-mCherry and XVpro-mCherry in Fig. 5. Infiltrated plants were kept in a growth chamber, under the same conditions as described above.

### Plasmid construction

Plasmids used in this study were constructed as follows. PCR was performed using KOD-Plus-Neo (TOYOBO, Osaka, Japan). Plasmids were isolated with the FastGene Plasmid Mini Kit (Nippon Genetics, Tokyo, Japan) and verified by sequencing (Eurofins Genomics). Primer sequences are listed in Supplementary Table S1.

The antibody-based probe construct, encoding the HA epitope-binding 12CA5-scFv antibody fused to mCherry (FB-mCherry), was generated by introducing the 15F11-HA-mCherry segment from pCMV-15F11-HA-mCherry (Addgene #129291) into the Agrobacterium binary vector pCAMBIA1301.1 (Komatsu et al. 2021). A 1.6 kb region spanning the HA antibody and mCherry coding sequences was PCR amplified using primers pC1301-1-SalI-15F11-HA-F and pC1301-1-BamHI-mCherry-R, and cloned into SalI/BamHI-digested pCAMBIA1301.1 using In-Fusion cloning (Takara Bio, Shiga, Japan).

PlAMV replicon construct PlAMV replicon-HA, encoding the replicase protein with a C-terminal HA epitope tag, was made by amplifying a 1.2 kb fragment spanning the 3′ end of the replicase-coding sequence with the HA tag sequence added using primers Li1-3073F and RdRp-HA-SpeI-R. The amplified fragment was cloned into the pLi1 (Ozeki et al. 2006) digested with BglII and SpeI. Li1-replicase-HA-GFP, GFP-expressing PlAMV vector with a C-terminal HA tag fused to the replicase, was generated by preparing 2 fragments, (i) a 1.1 kb region encoding C-terminal portion of a replicase added with an HA tag, amplified using primers Li1-3073F and Li1-RdRp-HA-R, and (ii) a 2.6 kb region encoding TGBp1-3, GFP, and CP sequences, amplified using primers Li1-HA-TGBp1-F and Li1-CP-R, both with Li1-GFP as a template (Minato et al. 2014), and cloned into BglII/SpeI-digested pLi1 using In-Fusion cloning.

Plasmids for expression of ER-localized GFP with an HA tag and 3 HA tags, ER-GFP-HA and ER-GFP-3HA, respectively, were derived from ER-gk (CD3-955, Arabidopsis Biological Resource Center (ABRC); Nelson et al. 2007 ). One HA and 3 HA tags were added before the C-terminal ER retention signal (HDEL) of ER-localized GFP. To construct these plasmids, 2 fragments were prepared: (i) an 820 base region encoding ER-targeted GFP lacking the C-terminal ER retention signal, amplified using primers pC1301-1-SalI-ER-GFP5-F and ER-GFP5-R with ER-gk as a template, and (ii) 2 synthesized fragments, encoding the C-terminal sequence of ER-GFP followed by HA or 3 HA tags, a linker sequence (NGAASE), and ER retention signal, designated as CterGFP-HA-HDEL or CterGFP-3HA-HDEL, respectively (Eurofins Genomics). The (i) and either of the 2 (ii) fragments, amplified using CterGFP-HA-HDEL-F and pC1301-1-BamHI-HA-HDEL-R, were combined by overlap extension PCR using the primer set pC1301-1-SalI-ER-GFP5-F and pC1301-1-BamHI-HA-HDEL-R, and cloned into SalI/BamHI-digested pCAMBIA-1301.1-sGFP.

Two-component system constructs are composed of a responder vector XVpro-FB-mCherry or XVpro-mCherry, and an activator virus Li1-XV or Li1-replicase-HA-XV. XVpro-FB-mCherry was generated by introducing the 15F11-HA-mCherry segment from pCMV-15F11-HA-mCherry into the vector pMDC221 (Brand et al. 2006). A 1.6 kb region spanning the HA antibody and mCherry coding sequences was amplified by PCR using primers pMDC221-AscI-antiHA-mCherry-F and pMDC221-PacI-antiHA-mCherry-R and cloned into AscI/PacI-digested pMDC221 using In-Fusion cloning. XVpro-mCherry was generated by amplifying a 700 base mCherry coding sequence using primers pMDC221-AscI-mCherry-F and pMDC221-PacI-antiHA-mCherry-R and cloned into AscI/PacI-digested pMDC221.

A vector containing XV transcriptional activator, pART27-35Sa-XV-DHA, was generated as follows. XVE coding sequence was amplified from pMDC150 (Brand et al. 2006) using primers XVE-F01 and XVE-R01, and cloned into pENTR/D-TOPO (Thermo Fisher Scientific, Carlsbad, CA, USA) to make pENTR-XVE_no_stop_codon. Using this pENTR-XVE_no_stop_codon as a template, inverse PCR was performed using primers pENTR-F01 and XVE_V_R01, and the amplified fragment was self-ligated to make pENTR-XV_no_stop_codon. pART27-Sa-XV-DHA was generated by LR reaction between pENTR-XV_no_stop_codon and pART27-35Sa-GWB-DHA (Ogata et al. 2012).

Li1-XV containing the XV transcriptional activator was constructed by amplifying 2 fragments: (i) a sequence fragment ranging from the C-terminal 1 kb region of the replicase to TGBp3 of PlAMV using primers Li1-3197F and Li1-TGBp3-XV-R, and (ii) the XV coding region from pART27-35Sa-XV-DHA using primers XV-F and XV-MluI-R. These 2 fragments were cloned into BglII/MluI-digested Li1-GFP. Li1-replicase-HA-XV, which harbors a C-terminal HA tag on the replicase in Li1-XV, was generated by amplifying a 2.2 kb fragment from Li1-replicase-HA-GFP using primers Li1-3197F and Li1-TGBp3-XV-R, and cloned into BglII/MluI-digested Li1-GFP with the PCR fragment of the XV coding region.

### Confocal microscopy

Agroinfiltrated *N. benthamiana* leaf epidermal cells were imaged using a Nikon AX/AX R confocal laser scanning microscope. Aniline blue (405 nm), GFP (488 nm), and mCherry (561 nm) were excited using laser diodes, and detected within the ranges of 429 to 474 nm, 509 to 541 nm, and 579 to 620 nm, respectively. All fluorescence images were processed using NIS-Elements AR software (Nikon). Fluorescence intensities were quantified using ImageJ/Fiji software (https://imagej.net/software/fiji) by analyzing GFP and mCherry fluorescence signals with Plot Profile. For callose staining, leaves of agroinfiltrated *N. benthamiana* were infiltrated with 0.01% (w/v) aniline blue in 50 mM phosphate buffer (pH 7.0) approximately 30 min before imaging.

### RNA extraction and northern blot analysis

Total RNA from agroinfiltrated leaves was extracted at 24, 36, 48, and 72 hpi using the NucleoSpin RNA Plant kit (Takara Bio). Northern blotting was performed as described previously (Komatsu et al. 2011, 2021). Digoxigenin-labeled RNA probe targeting viral CP was described previously (Ozeki et al. 2009). RNA probe used for the detection of negative-strand viral RNA was transcribed from cDNA of the 5′ untranslated region and the replicase gene (bases 1 to 920), which was amplified with the primer set T7-Li1-1F and LiPr-1006R.

### Protein extraction, SDS-PAGE, and immunoblotting analysis

Total protein was extracted from agroinfiltrated leaves at 2 dpi and fractionated into soluble (S) and membrane-enriched pellet (P) fractions as previously reported (Komatsu et al. 2021; Shinji et al. 2023). Proteins were separated by SDS-PAGE using 12.5% e-PAGEL HR gels (ATTO, Tokyo, Japan) or 3% to 8% Tris-acetate NuPAGE gels (Thermo Fisher Scientific) and analyzed by immunoblotting with following antibodies: α-PlAMV CP IgG (1:1,000, Ozeki et al. 2006), α-mCherry IgG (1:800, GTX59788, GeneTex, Irvine, CA, USA), and α-PlAMV replicase antibody (1:1,000, Komatsu et al. 2011) were used as primary antibodies, and anti-rabbit IgG, HRP-Linked Whole Ab Donkey (1:1,000, lot. 17824033, Cytiva, Tokyo, Japan) was used as secondary antibody.

## Supplementary Material

kiaf240_Supplementary_Data

## Data Availability

All relevant biological materials are available from the corresponding author upon request. Supporting data are available in the Supplementary data published online.

## Supplementary data

The following materials are available in the online version of this article.

**Supplementary Figure S1.** Confocal images of the subcellular localization of GFP, expressed from Li1-replicase-HA-GFP or Li1-GFP, and FB-mCherry in *Nicotiana benthamiana* leaves.

**Supplementary Figure S2.** Northern blotting analysis of the accumulation of PlAMV genomic and subgenomic RNAs in *Nicotiana benthamiana* leaves co-expressing XVpro-FB-mCherry with Li1-replicase-HA-XV or Li1-XV at 24, 36, and 48 hpi.

**Supplementary Table S1.** Names and sequences of primers used in this study.

## Dive Curated Terms

The following phenotypic, genotypic, and functional terms are of significance to the work described in this paper:

CLSM Gramene: Confocal Laser-Scanning Microscopy
CLSM Araport: Confocal Laser-Scanning Microscopy
