# Supplementary material for: Live-cell imaging of a plant virus replicase during infection using a genetically encoded, antibody-based probe
Source: Plant Physiol. 2025 Jun 6;198(2):kiaf240. doi: 10.1093/plphys/kiaf240 (PMC12205435; doi:10.1093/plphys/kiaf240)
Supplement: kiaf240_Supplementary_Data [file kiaf240_supplementary_data.pdf]

Supplementary Data

Live-cell imaging of a plant virus replicase during infection using a genetically encoded, antibody-based probe

Chiyusa Ishihara[1], Nobumitsu Sasaki[1,2], Yasuhiko Matsushita[1], Tomohiro Tsunoda[1], Tsutomu Arie[1,2], Richard S. Nelson[3], Ken Komatsu[1,2,*]

1 Graduate School of Agriculture, Tokyo University of Agriculture and Technology (TUAT), Fuchu, Tokyo 183-8509, Japan

2 Institute of Global Innovation Research (GIR), Tokyo University of Agriculture and Technology (TUAT), Fuchu, Tokyo 183-8509, Japan

3 Department of Entomology and Plant Pathology, Oklahoma State University, Stillwater, Oklahoma, USA

List of Supplementary Data

1. Supplementary Figure S1. Confocal images of the subcellular localization of GFP, expressed from Li1-replicase-HA-GFP or Li1-GFP, and FB-mCherry in *Nicotiana benthamiana* leaves.
2. Supplementary Figure S2. Northern blotting analysis of the accumulation of PlAMV genomic and subgenomic RNAs in *Nicotiana benthamiana* leaves co-expressing XVpro-FB-mCherry with Li1-replicase-HA-XV or Li1-XV at 24, 36, and 48 hours post-infiltration (hpi).
3. Supplementary Table S1. Names and sequences of primers used in this study.

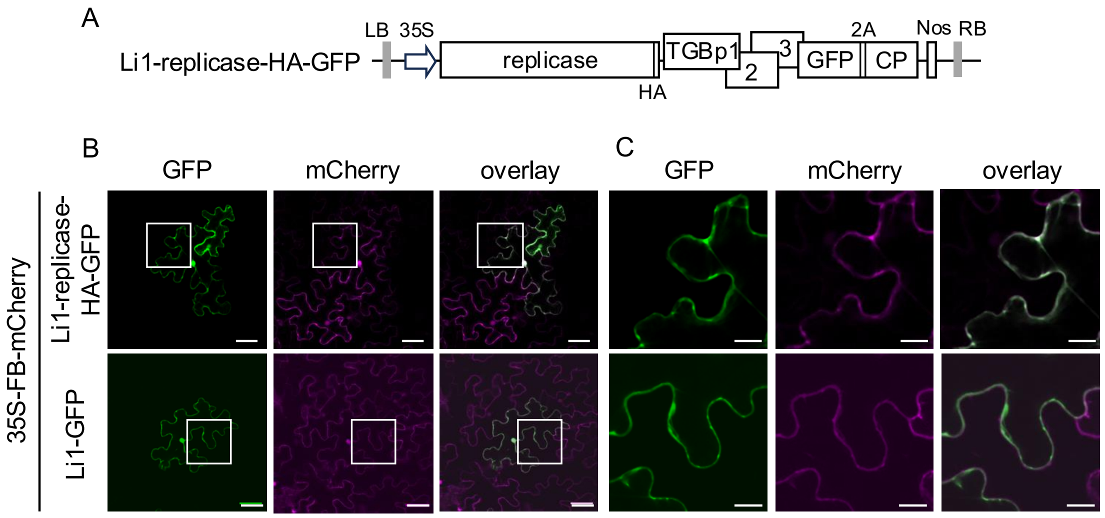

Supplementary Figure S1. Confocal images of the subcellular localization of GFP (green), expressed from Li1-replicase-HA-GFP or Li1-GFP, and FB-mCherry (magenta) in *Nicotiana benthamiana* leaves. Li1-replicase-HA-GFP, Li1-GFP, and FB-mCherry were expressed under the control of the cauliflower mosaic virus 35S promoter. Images were captured at 48 hours post-infiltration (hpi). A) shows schematic representations of the construct Li1-replicase-HA-GFP. See Fig. 2A legend for details. B) shows transverse sections of whole epidermal cells, and C) shows magnified images of the area in the white squares in B). Scale bars, 50 µm (B) and 10 µm (C).

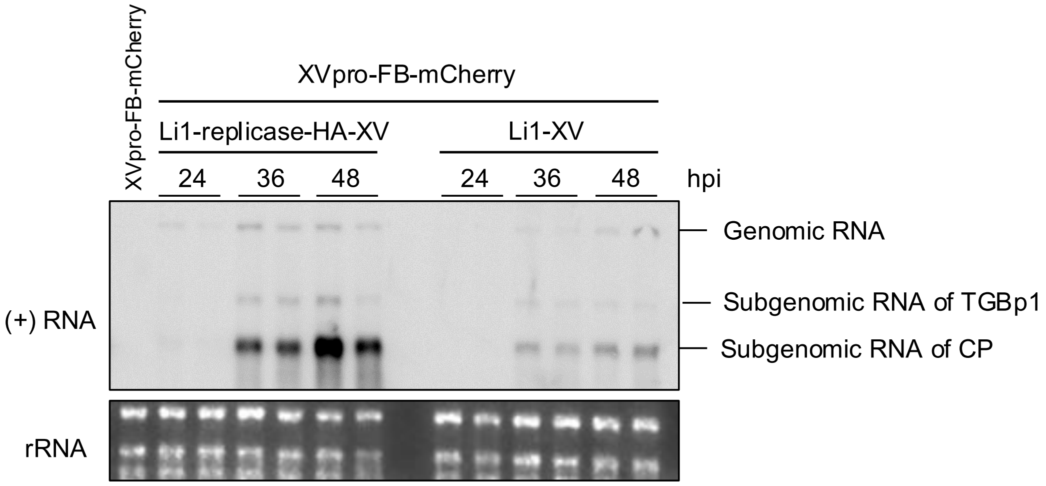

Supplementary Figure S2. Northern blotting analysis of the accumulation of PlAMV genomic and subgenomic RNAs in *Nicotiana benthamiana* leaves co-expressing XVpro-FB-mCherry with Li1-replicase-HA-XV or Li1-XV at 24, 36, and 48 hours post-infiltration (hpi). Approximately 1 µg of total RNA was loaded and DIG-labeled RNA probe targeting the CP region of the positive strand of PlAMV (Ozeki et al. 2009) was used. The result shown is representative of two independent experiments. Ethidium bromide-stained ribosomal RNA (rRNA) is shown as a loading control.

Supplementary Table S1. Names and sequences of primers used in this study.

Primer name	Sequence (5' - 3')	Purpose
pC1301-1-SalI-15F11-HA-F	CTTGACCATGGTCGACATGGCCGAGGTGAAG CT	Amplication of FB-mCherry for In-Fusion cloning
pC1301-1-BamHI-mCherry-R	CCTAGGTACCGGATCTTACTTGTACAGTCGTC CATGCC	Amplication of FB-mCherry for In-Fusion cloning
Li1-3073F	CCATCAATGCACGCCTGACC	Amplication of replicase for In-Fusion cloning
RdRp-HA-SpeI-R	CTTTCCACGGTTCTAGACTAGTTCACGCATAG TCAGGAACATCGTATGGGTAATCGGAGTTAAG GAAAGATGAACC	Amplication of replicase for In-Fusion cloning
Li1-RdRp-HA-R	CATAGTCAGGAACATCGTATGGGTAATCGGAG TTAAGGAAAGATGAACC	Amplication of replicase for In-Fusion cloning
Li1-HA-TGBp1-F	CGATGTTCCTGACTATGCGTGAGATTAACATG GACATAGTCATCTCAGC	Amplication of TGBp, GFP and CP for In-Fusion cloning
Li1-CP-R	TCTTTCCACGGTTCTAGACTAGT	Amplication of TGBp, GFP and CP for In-Fusion cloning
pC1301-1-SalI-ER-GFP5-F	CTTGACCATGGTCGAATGAAGGTACAGGAGG GTTTGTTC	Amplication of ER-GFP for In-Fusion cloning
ER-GFP5-R	AGATCTGTATAGTTCATCCATGCCATG	Amplication of ER-GFP for In-Fusion cloning
CterGFP-HA-HDEL-F	ATGGATGAACTATACAGATCTTACCCA	Amplication of HA-HDEL for In-Fusion cloning
pC1301-1-BamHI-HA-HDEL-R	CCTAGGTACCGGATCTTACAGCTC	Amplication of HA-HDEL for In-Fusion cloning
pMDC221-AscI-antiHA-mCherry-F	TAGCCTCGAGGCGCGCCATGGCCGAGGTGAA GC	Amplication of FB-mCherry for In-Fusion cloning
pMDC221-PacI-antiHA-mCherry-R	TGGATCGACTAGTTAATTAATTACTTGTACAGC TCGTCCATGCC	Amplication of FB-mCherry for In-Fusion cloning
pMDC221-AscI-mCherry-F	TAGCCTCGAGGCGCGCCATGGTGAGCAAGGG CGAGGAGG	Amplication of mCherry for In-Fusion cloning
XVE-F01	CACCATGAAAGCGTTAACGGCCAGGC	Amplication of XVE for TOPO cloning
XVE-R01	GACTGTGGCAGGGAAACCCTCTGCC	Amplication of XVE for TOPO cloning
pENTR-F01	AAGGGTGGGCGCGCCGACCCAGC	Inverse PCR amplication of XV only vector
XVE_V_R01	ATCCCCACCGTACTCGTCAATTCC	Inverse PCR amplication of XV only vector
Li1-3197F	ACCGGACTTCTCCGAAGATCTC	Amplication of replicase and TGBp for In-Fusion cloning
Li1-TGBp3-XV-R	ACGCTTTCATCGGATCCTCAGAGGTCGG	Amplication of replicase and TGBp for In-Fusion cloning
XV-F	TGAGGATCCGATGAAAGCGTTAACGGCCAGG	Amplication of XV for In-Fusion cloning
XV-MluI-R	AAGAAGGTCAAAATTACGCGTATCCCCACCGT ACTCGTC	Amplication of XV for In-Fusion cloning
T7-Li1-1F	CTAATACGACTCACTATAGAAAACAAACCTAC A CAAACCAA	RNA probe for Northern blotting
LiPr-1006R	TTGACGGACTTAATGTAGAAGA	RNA probe for Northern blotting
